# P-2049. Interim Estimates of Effectiveness of Updated 2023-2024 (Monovalent XBB.1.5) COVID-19 Vaccines Against COVID-19-Associated Outcomes Among Medicare Enrollees with End Stage Renal Disease — United States, September – December 2023

**DOI:** 10.1093/ofid/ofae631.2205

**Published:** 2025-01-29

**Authors:** Amanda B Payne, Shannon Novosad, Ryan E Wiegand, Morgan Najdowski, Danica J Gomes, Megan Wallace, Alia Bayatti, Heng-Ming Sung, Yue Zhang, Bradley Lufkin, Yoganand Chillarige, Ruth Link-Gelles

**Affiliations:** CDC, Atlanta, Georgia; Centers for Disease Control and Prevention; Centers for Disease Control and Prevention; CDC, Atlanta, Georgia; Centers for Disease Control and Prevention; CDC, Atlanta, Georgia; CMS, DC, District of Columbia; Acumen LLC, Burlingame, California; Acumen LLC, Burlingame, California; Acumen LLC, Burlingame, California; Acumen LLC, Burlingame, California; Centers for Disease Control and Prevention

## Abstract

**Background:**

Persons with end stage renal disease (ESRD) on maintenance dialysis are at high risk for severe COVID-19 disease. On September 12, 2023, updated 2023-2024 COVID-19 vaccination was recommended in the United States for all persons aged ≥6 months. Due to the innate immune dysfunction and high prevalence of additional underlying conditions, including immunocompromising conditions (ICs), among individuals with ESRD, there is concern about reduced vaccine effectiveness (VE). Understanding the VE of updated doses among persons with ESRD will inform the need for additional doses in this population.

Table 1

**Figure:**
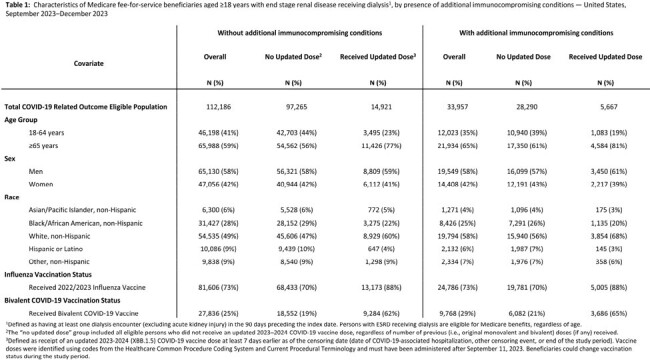

Characteristics of Medicare fee-for-service beneficiaries aged ≥18 years with end stage renal disease receiving dialysis1, by presence of additional immunocompromising conditions — United States, September 2023–December 2023

**Methods:**

A retrospective cohort study was conducted among Medicare fee-for-service beneficiaries aged ≥18 years with ESRD receiving dialysis using Medicare enrollment and claims records. Follow-up began on September 17, 2023, and continued until the earliest occurrence of a censoring event, COVID-19–associated outcome (medically attended COVID-19 or COVID-19-associated hospitalization), or study end. A marginal structural Cox model was used to estimate VE (calculated as [1 – hazard ratio]*100%), interpreted as the benefit of an updated COVID-19 vaccine dose compared with no updated dose, by presence of additional ICs.

Table 2

**Figure:**
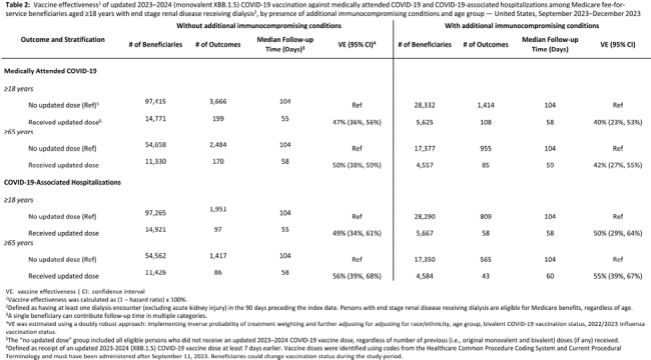

Vaccine effectiveness1 of updated 2023–2024 (monovalent XBB.1.5) COVID-19 vaccination against medically attended COVID-19 and COVID-19-associated hospitalizations among Medicare fee-for-service beneficiaries aged ≥18 years with end stage renal disease receiving dialysis2, by presence of additional immunocompromising conditions and age group — United States, September 2023–December 2023

**Results:**

During September 17 – December 30, 2023, 14,921/112,186 (15%) Medicare beneficiaries aged ≥18 years with ESRD without additional ICs and 5,667/33,957 (17%) beneficiaries aged ≥18 years with ESRD and additional ICs received an updated 2023-2024 COVID-19 vaccine dose (Table 1). Among those with ESRD without additional ICs, VE against medically attended COVID-19 was 47% (95% confidence interval [CI]: 36% - 56%) and against COVID-19-associated hospitalization was 49% (95% CI: 34% - 61%). Among those with ESRD and additional ICs, VE against medically attended COVID-19 was 40% (95% CI: 23% - 53%) and against COVID-19-associated hospitalization was 50% (95% CI: 29% - 64%)(Table 2).

**Conclusion:**

VE among adults with ESRD was similar to published updated 2023-2024 COVID-19 VE estimates among the general adult population. These data support the recommendation that adults with ESRD get an updated COVID-19 vaccine, especially people 65 years or older and people with ICs, who are eligible for additional doses.

**Disclosures:**

Ryan E. Wiegand, PhD, Merck & Co, Inc.: Stocks/Bonds (Public Company)|Sanofi ADR: Stocks/Bonds (Public Company) Heng-Ming Sung, MPH, CDC: Author is an employee of Acumen LLC. This study was funded through an inter-agency agreement between CDC and CMS for which Acumen is a contractor. Yue Zhang, MS, CDC: Author is an employee of Acumen LLC. This study was funded through an inter-agency agreement between CDC and CMS for which Acumen is a contractor. Bradley Lufkin, MPA, CDC: Author is an employee of Acumen LLC. This study was funded through an inter-agency agreement between CDC and CMS for which Acumen is a contractor. Yoganand Chillarige, MPA, CDC: Author is an employee of Acumen LLC. This study was funded through an inter-agency agreement between CDC and CMS for which Acumen is a contractor.

